# Cellular Stress Assay in Peripheral Blood Mononuclear Cells: Factors Influencing Its Results

**DOI:** 10.3390/ijms232113118

**Published:** 2022-10-28

**Authors:** Belay Tessema, Janine Riemer, Ulrich Sack, Brigitte König

**Affiliations:** 1 Institute of Clinical Immunology, Faculty of Medicine, University of Leipzig, 04103 Leipzig, Germany; 2 Department of Medical Microbiology, College of Medicine and Health Sciences, University of Gondar, Gondar 196, Ethiopia; 3 Magdeburg Molecular Detections GmbH & Co. KG, 39104 Magdeburg, Germany; 4 Institute of Medical Microbiology and Virology, Faculty of Medicine, University of Leipzig, 04103 Leipzig, Germany

**Keywords:** cellular stress assay, peripheral blood mononuclear cells, mitochondrial function

## Abstract

Cellular stress is central to the understanding of pathological mechanisms and the development of new therapeutic strategies and serves as a biomarker for disease progression in neurodegeneration, diabetes, cancer, cardiovascular and other chronic diseases. The common cellular stress assay (CSA) based on Seahorse technology in peripheral blood mononuclear cells (PBMCs) shows inconsistent results, which prevents its use as a biomarker for the progression of chronic diseases. Therefore, the aim of this study was to investigate potential factors that affect the CSA in PBMCs. We measured the CSA parameters in PBMCs from study participants and compared the results according to the potential factors, namely, the PBMC isolation method, age, seasonal variation and the gender of the study participants. PBMCs were isolated by OptiPrep^®^ and Robosep^TM^-S methods. PBMCs isolated with the OptiPrep method showed much higher extracellular acidification and higher respiration compared to Robosep-isolated cells. Moreover, OptiPrep-isolated cells showed a higher number of outliers for the proton production rate (PPR) and a high respiratory quotient, indicating impurities with other cells, such as platelets, and technical inconsistencies. PBMCs from older individuals showed higher maximal respiration, spare capacity and extracellular acidification than younger participants. Additionally, in winter, maximal respiration and spare capacity decreased. From spring until early autumn, spare capacity and maximal respiration continuously increased. Elderly males also showed higher basal respiration, spare capacity and extracellular acidification than females. In conclusion, the findings of this study clearly demonstrate that the results of CSA parameters measured in PBMCs are influenced by the PBMC isolation method, age, seasonal variation and gender. Therefore, we recommend that researchers and physicians properly interpret the results of CSA parameters in PBMCs by considering these factors. It is important to use separate CSA evaluation standards based on the isolation method, age, gender and season-dependent factors. To assess the cellular stress situation in PBMCs, both extracellular acidification and mitochondrial respiration should be taken into account. Further study of additional factors, such as mitochondrial mass, should be conducted to improve the measurement of CSA parameters for the assessment of the real mitochondrial fitness.

## 1. Introduction

Chronic diseases due to dysfunctional metabolism are a major healthcare challenge in the developed world [1,2]. The contemporary sedentary lifestyle and the consumption of low-cost high-calorie foods are major risk factors for chronic diseases, which increasingly challenge the healthcare system, especially in terms of diagnosis and management [3].

Mitochondria are a target and source of oxidative stress, and the failure to maintain normal mitochondrial function with the appropriate balance of autophagy and biogenesis eventually causes cell death and/or cellular senescence [4,5,6]. Many chronic diseases are associated with both increased mitochondrial dysfunction and systemic oxidative stress [1,4,6,7]. These findings have resulted in the hypothesis that circulating peripheral blood mononuclear cells (PBMCs) can serve as bioenergetic biomarkers of systemic exposure to pro-inflammatory cytokines or metabolic stressors [8,9,10]. A previous study reported that human PBMCs isolated from Type 2 diabetic patients have changes in mitochondrial mass, mitochondrial morphology and membrane potential [11]. These findings imply that the measurement of cellular bioenergetics in leukocytes can be used as a surrogate marker for mitochondrial function in several pathologies, including Alzheimer’s disease and autoimmune diseases [12,13,14].

The findings of previous studies also support the emerging concept that circulating leukocytes can serve as predictive biomarkers of mitochondrial function under conditions of metabolic stress [15,16,17,18]. These studies prompted researchers to begin an integrated approach using PBMCs isolated from human blood to establish a quantitative assay of mitochondrial function that will have the power to predict disease progression and the response to treatment [19].

In the last couple of years, the diagnosis of mitochondrial dysfunctions has increased in health facilities [4,19,20,21,22,23,24]. Psychological and neurological disorders, such as autism, depression, burn-out or Alzheimer’s disease, and diseases such as diabetes or cancer are linked to mitochondrial dysfunctions [24,25]. Nevertheless, mitochondrial dysfunctions are primarily defined as mutations within genes encoded within mitochondria and the nucleus. In contrast, mitochondrial malfunctions beyond the general classification were often ignored in the past. Since the development of the Seahorse technology for the cellular stress assay (CSA), a large number of studies have been published on approaches to mitochondrial function assessment in terms of oxygen consumption and extracellular acidification [4,19,22,23,26].

Various studies have shown that some parameters of the Seahorse-based CSA, such as proton leak and spare capacity, are clear indicators of mitochondrial function [4,23]. Moreover, it is often suggested that PBMCs have a high potential to measure CSA parameters for the assessment of mitochondrial function in patients as a whole [27,28,29,30,31,32,33,34,35,36,37,38,39]. However, other studies have shown that the use of PBMCs to measure CSA parameters to assess mitochondrial function is inconsistent. For instance, some reports have found correlations between PBMCs’ mitochondrial health and muscle strength in elderly persons [27]. In contrast, another study showed that PBMCs do not reflect skeletal muscle mitochondrial function [20]. Consequently, the measurement of CSA parameters is much more difficult to interpret than expected and has shown inconsistent results.

A previous review by Betsou and his colleagues documented the potential effects of PBMC isolation methods on the results of different assays. The critical preanalytical factors during PBMC isolation are the isolation method (density gradient or magnetic sorting), the use of a barrier, possible red blood cell lysis and the centrifuge type [30]. The potential influence of age on the results of the CSA is also highlighted by previous reports, as aging is the progressive loss of tissue and organ function over time. This effect might be correlated with an increased demand for cellular repair mechanisms and, potentially, the higher generation of reactive oxygen species (ROS) due to old age or less efficient ATP production [31,32]. Seasonal variation was also associated with variations in the occurrence of diseases [33]. Furthermore, people show better physical performance during spring and summer and weaker performance during winter [34]. These variations could have an influence on the results of CSA parameters. Moreover, gender differences in the level of oxidative stress were reported in a previous study. Females who are premenopausal appear to have lower levels of oxidative stress compared to men. One reason for this apparent gender difference could be due to the antioxidant properties of estrogen. Moreover, antioxidant enzyme activity levels, NADPH-oxidase levels and angiotensin II may also play important roles [35]. It is also believed that women have a higher life expectancy [36]. Based on these lines of evidence, we hypothesize that the results of CSA parameters in PBMCs could be influenced by factors including the PBMC isolation method, age, seasonal variation and gender. To the best of our knowledge, the direct effects of these potential factors on the results of CSA parameters in PBMCs have not been studied. Therefore, the aim of this study was to investigate the potential factors that influence the measurements of CSA parameters in PBMCs, including the PBMC isolation method, age, seasonal variation and the gender of the individual.

## 2. Results

### 2.1. Characteristics of the Study Participants According to the Potential Factors Considered

Of the total 518 study participants, 286 persons were females and 232 were males. Of the total 493 patients, PBMCs from 289 patients were Opti-isolated, while in 204 patients, PBMCs were Rob-isolated. Similarly, in 13 of the 25 healthy individuals, PBMCs were Opti-isolated, and PBMCs from 12 individuals were Robo-isolated. In the majority of participants (478 of the 518), CSA parameters were analyzed using a Seahorse XFp Analyzer, and in the remaining 40 participants, CSA parameters were analyzed by a Seahorse XF96 Analyzer. Only 9 of the Opti-isolated and 31 of the Robo-isolated PBMCs were analyzed in the Seahorse XF96 Analyzer. CSA parameters measured by both Seahorse XFp and Seahorse XF96 analyzers revealed comparable results. Of the total 518 study participants, 145 participants were recruited in spring, 133 were recruited in summer, 112 were recruited in autumn, and 128 were recruited in winter. Among the total study participants, 61 were young, 128 were midlife adults, 143 were in mature adulthood, 112 were in late adulthood, and 74 were in the old-age group. The distribution of healthy individuals was comparable among groups (24% young, 32% midlife, 24% mature adults and 20% late adults); however, there were no healthy individuals in the old-age group.

### 2.2. Yields of PBMCs in Robosep^TM^-S and OptiPrep^®^ Isolation Methods

The measurement of CSA parameters requires the isolation of a sufficient number of PBMCs. For patients, it is important to lower the amount of blood they have to donate for CSA measurement. Moreover, the method of choice for PBMC isolation should be time-efficient. We therefore first compared the number of PBMCs that could be obtained by the OptiPrep^®^ and Robosep^TM^-S isolation methods. This study showed that the number of PBMCs isolated from whole blood was about 2.44 times higher in the case of the Robosep^TM^-S method compared to the OptiPrep isolation method. This result indicates that the isolation of PBMCs by the Robosep^TM^-S method requires collecting a lower amount of blood from patients and saves about 50% of the time required for PBMC isolation compared to the OptiPrep^®^ isolation method.

### 2.3. The Results of CSA Parameters in Robosep^TM^-S- and OptiPrep^®^-Isolated PBMCs

**Figure 1:**
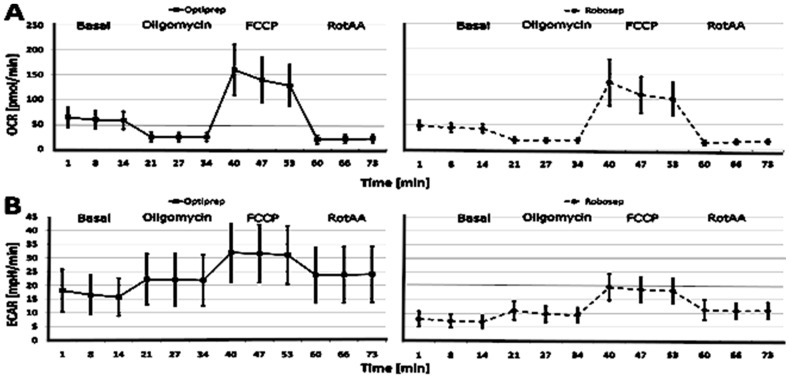
Cellular stress assay (CSA) with OptiPrep-isolated (*n* = 302) and Robosep-isolated PBMCs (*n* = 216). (**A**) Oxygen consumption rate (OCR) and (**B**) extracellular acidification rate (ECAR). The horizontal lines show the mean ± SD. FCCP = carbonyl cyanide 4-(trifluoromethoxy) phenylhydrazone; SD = standard division; RotAA = rotenone and antimycin A.

In this study, the CSA parameters, oxygen consumption rate (OCR) and extracellular acidification rate (ECAR) were analyzed in both Robosep^TM^-S- and OptiPrep^®^-isolated PBMCs (Table 1 and Figure 1). OCR was used as an indicator of oxidative phosphorylation, while ECAR was used as an indicator of the glycolytic conversion of glucose to lactate. As shown in Figure 1A, the basal OCR was measured before the sequential addition of oligomycin, FCCP, and antimycin A and/or rotenone, with the measurement of changes in OCR after each addition. The time course is annotated to show the relative contribution of non-respiratory chain oxygen consumption, ATP-linked oxygen consumption, the maximal OCR after the addition of FCCP and the spare (reserve) capacity of the cells. ECAR was measured to demonstrate the changes in the basal glycolytic rate and maximal glycolytic rate. As shown in Figure 1B, after the measurement of the basal ECAR, oligomycin was added, which generally increases the glycolytic rate in response to the loss of mitochondrial ATP production. The addition of antimycin A/rotenone further increases the ECAR, giving an index of lactate production occurring when all mitochondrial electron transport is inhibited.

As shown in Table 1, it can be concluded that the distribution of the CSA parameter results in Opti- and Robo-isolated PBMCs is approximately normal, as the Shapiro–Wilk test (W) ≈ 1, and the 95% CI does not cross zero. To determine whether differences in the results of CSA parameters between Opti- and Robo-isolated PBMCs exist, we performed a statistical analysis using Cohen’s d test. It clearly indicated that PBMC isolation methods led to differences in the results of CSA parameters, including proton leak in pmol/min and non-mitochondrial respiration in %. The most severe differences were in the parameters of ECAR and respiration (especially basal respiration). In general, OCR was higher in Opti-isolated PBMCs compared to Robo-isolated cells. Moreover, the variation in ECAR was much higher in the case of Opti-isolated PBMCs than in Robo-isolated cells. These findings imply that for the assessment of CSA parameters with Seahorse technology, it is important to use separate evaluation standards based on the isolation method of choice.

**Figure 2:**
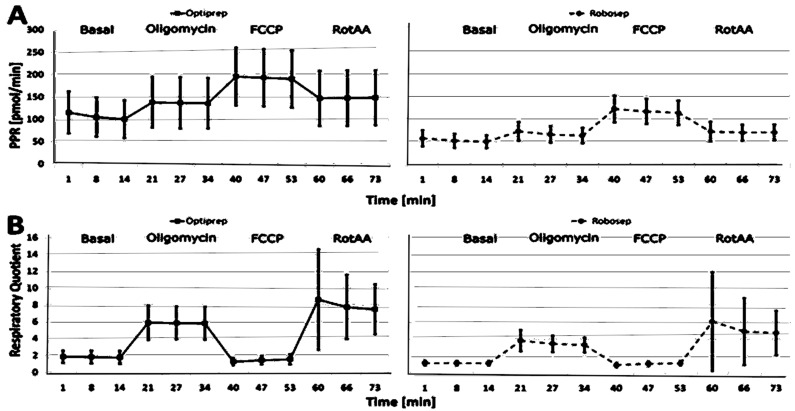
Cellular stress assay (CSA) with OptiPrep-isolated PBMCs (*n* = 293) and Robosep-isolated PBMCs (*n* = 185). (**A**) Proton production rate (PPR) and (**B**) respiratory quotient (RQ). The horizontal lines show the mean ± SD. It shows that the variation in proton production and extracellular acidification is much higher in Opti-isolated cells. FCCP = carbonyl cyanide 4-(trifluoromethoxy) phenylhydrazone; SD = standard division; RotAA = rotenone and antimycin A.

As the PBMC isolation methods showed a strong effect on ECAR, we further investigated the proton production rate (PPR), which allows the quantitative calculation of the glycolytic rate and therefore can be used to calculate the respiratory quotient (RQ) (Table 2 and Figure 2).

The RQ calculates the basal metabolic rate and indicates which macronutrients are being metabolized, as different energy pathways are used for fats, carbohydrates and proteins [37]. If metabolism in mitochondria consists solely of lipids, the respiratory quotient is approximately 0.7; for proteins, it is approximately 0.8, and for carbohydrates, it is 1.0 [21]. The more glucose consumed in the absence of mitochondria (glycolysis with concomitant lactate formation), the higher the RQ observed.

Robo-isolated PBMCs had an expected RQ of about 1.14 under basal conditions, and it was lower than 1.0 after the administration of FCCP. This indicates that no or only a little compensatory (anaerobic) glycolysis was present in Robo-isolated cells. In contrast, Opti-isolated cells had a much higher PPR and RQ (1.77 on average) under basal conditions. These findings indicate either the activation of Opti-isolated PBMCs or the presence of impurities. After randomly investigating potential contaminations using a Sysmex hemoanalyzer machine, we found platelets in Opti-isolated PBMCs but not in the case of Robo-isolated PBMCs. Moreover, the RQ of Robo-isolated PBMCs was approximately normal (Shapiro–Wilk test (W) ≈ 1). In contrast, the RQ of Opti-isolated PBMCs did not show a normal distribution (Shapiro–Wilk 0.71 to 0.76).

**Figure 3:**
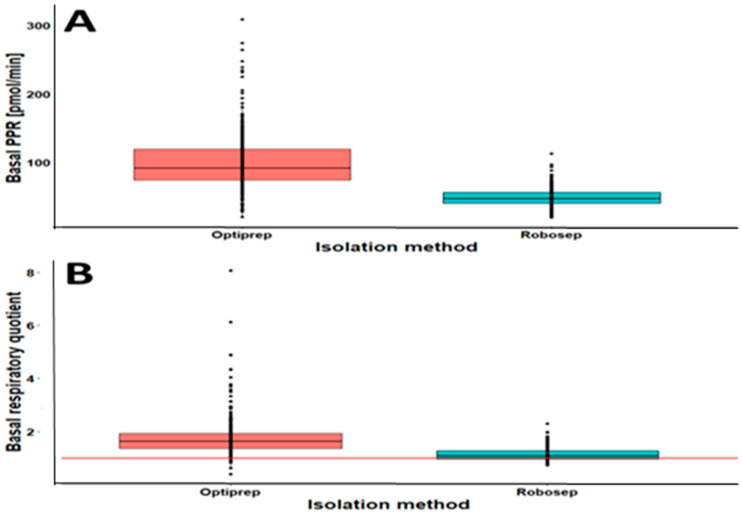
Boxplots of the basal proton production rate (PPR) (**A**) and the basal respiratory quotient (RQ) (**B**) of PBMCs isolated by OptiPrep^®^ (*n* = 293) density centrifugation or Robosep^TM^-S negative selection (*n* = 185). The red line in B indicates an RQ of 1.0, which is related to the complete oxidation of glucose by the cells. The outliers (black dots) in (**A**,**B**) are defined as data points for PPR and RQ, respectively, located outside the whiskers of the boxplots. The greater the number of outliers, the more inconsistent the PPR and RQ results.

These findings demonstrate that technical inconsistencies might be a problem in the case of the OptiPrep method of PBMC isolation. The inconsistency of the results in the case of Opti-isolated PBMCs is further affirmed by the observed outliers, as shown in Figure 3. The outliers in the PPR and RQ of Opti- and Rob-isolated PBMCs clearly demonstrate that the results of Robo-isolated PBMCs are more reliable and consistent than those of Opti-isolated PBMCs for Seahorse-based CSA analyses (Figure 3).

### 2.4. Younger Individuals Show Lower Oxygen Consumption and Lower Cellular Acidification

Metabolic changes that occur with age lead to the reduced performance and higher sensitivity of individuals to various health problems. In this study, we investigated the potential differences in CSA parameters between life phases. For this purpose, we defined five groups: young, midlife, mature adulthood, late adulthood, and old age. While analyzing various parameters of the CSA, we found spare capacity and maximal respiration to be the most interesting parameters in both Opti- and Robo-isolated PBMCs. As shown in Table 3, increasing trends of spare capacity and maximal respiration were observed with age in both Opti- and Robo-isolated PBMCs. These changes were more substantial in the case of Robo-isolated PBMCs. To verify the effect size, Cohen’s d analysis was performed for the young and old-age groups of study participants. The effect size was much higher in Robo-isolated PBMCs. Nevertheless, at least medium effects were observed for spare capacity (pmol/min) and maximal respiration (pmol/min) in Opti-isolated PBMCs.

**Figure 4:**
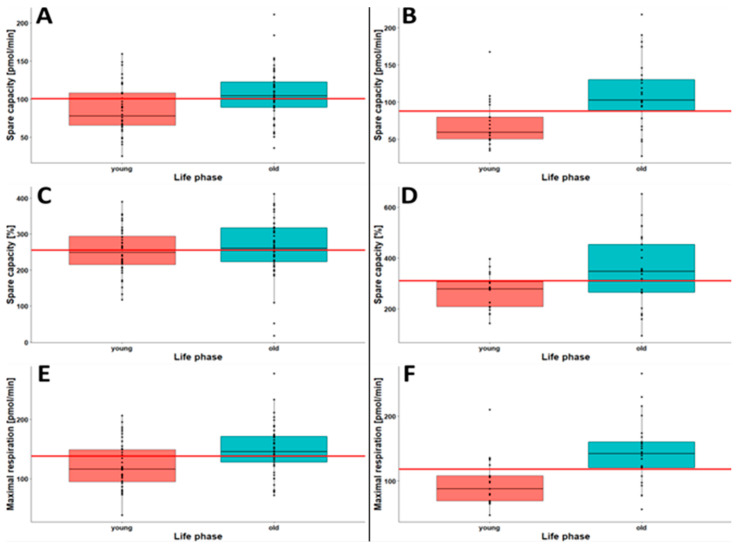
Boxplots of spare capacity (pmol/min) (**A**,**B**), spare capacity (%) (**C**,**D**) and maximal respiration (pmol/min) (**E**,**F**) in young and old persons in Opti-isolated PBMCs (**A**,**C**,**E**; young *n* = 40, old *n* = 49) and Robo-isolated PBMCs (**B**,**D**,**F**; young *n* = 21, old *n* = 25). The red horizontal line indicates the median of all life phases.

The differences in spare capacity and maximal respiration in young and old persons are presented as boxplots in Figure 4. General trends toward higher spare capacity and maximal respiration with the increasing age of the study participants were observed in both Opti-isolated PBMCs (Figure 4A,C,E) and Robo-isolated PBMCs (Figure 4B,D,F). In the case of Robo-isolated PBMCs, the medians of spare capacity and maximal respiration in young persons were below the medians of all life phases. In contrast, the medians in old persons were always above the medians of all life phases in Robo-isolated cells. This finding also holds true in the case of Opti-isolated PBMCs, except for spare capacity in %. Taken together, elderly persons generally showed higher spare capacity and maximal respiration than young persons. This effect might be correlated with an increased demand for cellular repair mechanisms and, potentially, the higher generation of reactive oxygen species (ROS) due to old age or less efficient ATP production [31,32].

**Figure 5:**
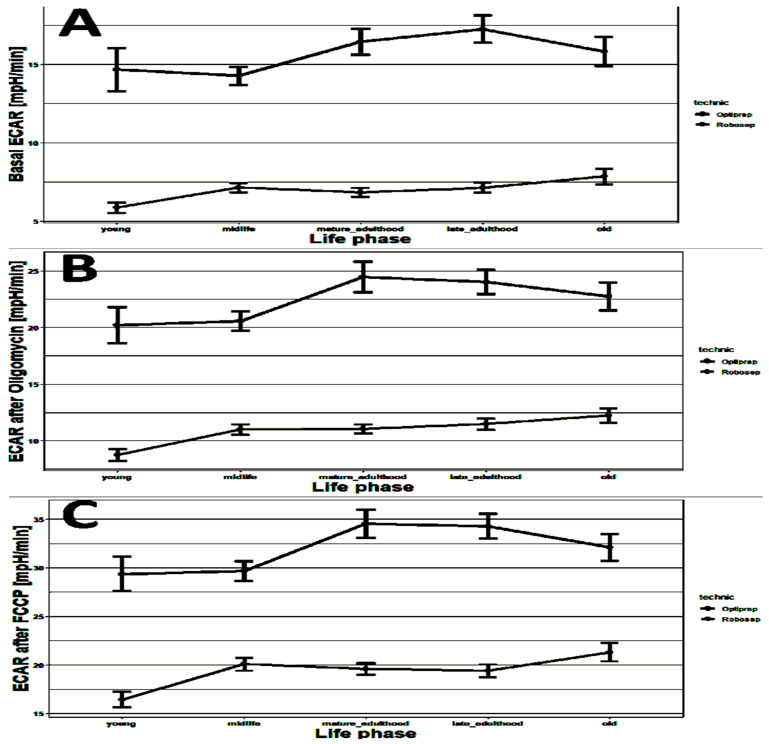
Changes in basal ECAR (mpH/min) (**A**), ECAR oligomycin (mpH/min) (**B**) and maximal ECAR (FCCP) (mpH/min) (**C**) with age observed in Opti-isolated PBMCs (black, *n* = 302) and Robo-isolated PBMCs (gray, *n* = 216). Young = 12 to 35 years; midlife = 35 to 50 years; mature adulthood = 50 to 60 years; late adulthood = 60 to 70 years; and old = 70 to 90 years. The horizontal lines show the mean ± SD.

As shown in Figure 5, the same trends of extracellular acidification were observed as in the case of spare capacity and maximal respiration in Figure 4 above. Higher extracellular acidification indicates the higher effort of the cell to achieve the required level of ATP via substrate-level phosphorylation from glycolysis independently of the mitochondria. The fact that mitochondrial respiration increases in a comparable manner indicates that either a much higher ATP demand is present in elderly persons or the ATP-generating mechanisms are inefficient.

**Figure 6:**
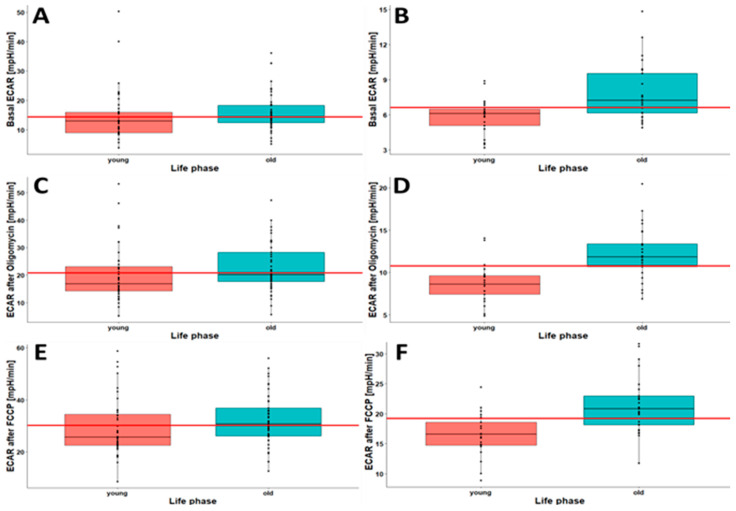
Boxplots of basal extracellular acidification rate (ECAR) (mpH/min) (**A**,**B**), ECAR oligomycin (mpH/min) (**C**,**D**) and maximal ECAR (FCCP) [mpH/min] (**E**,**F**) in young and old persons in Opti-isolated PBMCs (**A**,**C**,**E**; young *n* = 40, old *n* = 49) and Robo-isolated PBMCs (**B**,**D**,**F**; young *n* = 21, old *n* = 25). The red line indicates the median of all life phases.

As shown in Figure 6, in both Opti- and Robo-isolated PBMCs, a higher extracellular acidification rate was observed in the old-age group than in young individuals. This is interesting because the assessment of extracellular acidification in Opti-isolated PBMCs was often inadequate, which eventually led to only small effect sizes (Table 4).

The median of extracellular acidification in the Robo-isolated PBMCs from young persons was lower than the median of all life phases. However, the median of extracellular acidification in old persons was always higher than the median of all life phases. Strong effects were detectable in Robo-isolated PBMCs using Cohen’s d (Table 4). Taken together, the present findings show significant differences in CSA parameters in PBMCs between life phases. Robo-isolated PBMCs showed the greatest effects, and Opti-isolated PBMCs confirmed this trend.

### 2.5. Oxygen Consumption Rate and Extracellular Acidification Rate Show Seasonal Variation

**Figure 7:**
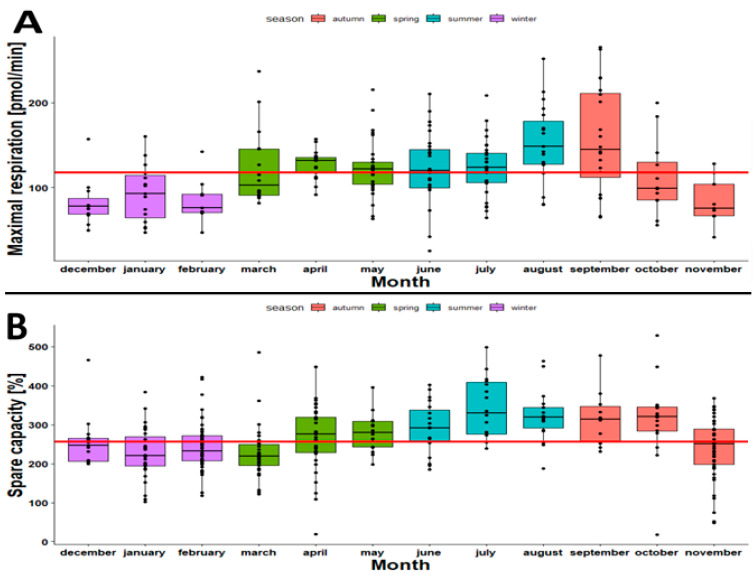
(**A**) Boxplots of maximal respiration (pmol/min) of Robo-isolated PBMCs by month. Sample size (*n*): January = 17, February = 7, March = 16, April = 17, May = 29, June = 29, July = 30, August = 19, September = 17, October = 12, November = 9 and December = 11. (**B**) Boxplots of spare capacity (pmol/min) of Opti-isolated PBMCs by month. Sample size (*n*): January = 31, February = 47, March = 16, April = 33, May = 16, June = 19, July = 18, August = 18, September = 10, October = 16, November = 45 and December = 15.

As we observed differences in bioenergetic performance with age, it was of interest to assess the effect of seasonal variations on the oxygen consumption rate (OCR) in PBMCs. In central Europe, there are four seasons: spring, summer, autumn and winter. Depending on the season, the occurrence of diseases varies [33]. Furthermore, people show better physical performance during spring and summer and weaker performance during winter [34]. Here, we investigated the effect of seasonal variations on OCR. The maximal respiration reached its peak level during August and September and then declined in autumn and winter in Robo-isolated PBMCs. Similar trends were observed in the case of spare capacity in Opti-isolated PBMCs (Figure 7).

To shed further light on the potential influence of cold and warm periods on mitochondrial function, we separated our data set into two groups. The warm group encompasses the months of June, July and August. The cold group includes the months of November, December, January and February. The results of the comparison of warm and cold periods of the year are shown in Table 5.

In both Opti- and Robo-isolated PBMCs, higher values of the following CSA parameters were observed in warmer periods compared to colder ones: proton leak (pmol/min and %), maximal respiration, spare capacity (pmol/min and %) and the Bioenergetic Health Index (BHI). All of these parameters are potential markers of higher mitochondrial activity, even in the case of proton leak [4,21,38]. Nevertheless, only spare capacity in % showed acceptable effects sizes in Opti-isolated PBMCs (d = 1.14, strong effect) and Robo-isolated PBMCs (d = 0.62, medium effect). The RQ was strongly affected by the addition of FCCP in Opti- and Robo-isolated PBMCs. In Robo-isolated PBMCs, the RQ after FCCP increased from 0.88 to 1.32 in winter, and in Opti-isolated PBMCs, it increased from 1.02 to 1.42. Taken together, our findings suggest that mitochondrial function is better in summer and declines during the winter period.

### 2.6. Males Show Higher Oxygen Consumption and Extracellular Acidification Rates Than Females

Because of the fact that women have a higher life expectancy [38], we investigated potential differences between males and females. The main differences observed between males and females were in basal respiration and spare capacity in %. The basal respiration in males increased in mature adulthood (50 to 60 years) in Opti-isolated PBMCs until old age (70 to 90 years). In Robo-isolated PBMCs, higher basal respiration was solely detectable in older males but showed a strong effect (Cohen’s d = 0.93). Opti-isolated PBMCs of older males also showed a medium effect in comparison to old women (Cohen’s d = 0.55). A comparable trend was also observed in the case of spare capacity in %. Old women showed a higher relative spare capacity than old males. This effect occurred in both Opti- and Robo-isolated PBMCs (medium effect sizes: Cohen’s d for Opti-isolated PBMCs = 0.53; Cohen’s d for Robo-isolated PBMCs = 0.71). These observations imply that spare capacity is a potential indicator of longevity. Based on the results of our study, the assessment of extracellular acidification in Opti-isolated PBMCs is inadequate. Therefore, we focused on Robo-isolated PBMCs to examine variations in extracellular acidification due to gender-related differences.

Males tended to have higher extracellular acidification than females beginning in mature adulthood (50 to 60 years). The largest difference was found in late adulthood (60 to 70 years). Even though old males had a comparable basal RQ to old females, the extracellular acidification rate constantly increased with age in males. The RQ after the administration of FCCP declined in both males and females from mature adulthood to old age. The effect size of males versus females in late adulthood was strong for basal PPR (Cohen’s d = 0.81) and basal RQ (Cohen’s d = 1.04) and medium in the case of maximal RQ after adding FCCP (Cohen’s d = 0.78). These findings indicate that the age between 50 and 70 is a critical life phase during which mitochondrial function declines, especially in males. In addition to spare capacity/maximal respiration, extracellular acidification is a potential indicator of senescence.

## 3. Discussion

In this study, we investigated the Seahorse-based CSA parameters in PBMCs and potential factors that might influence the results of CSA parameters, including the PBMC isolation method, age, seasonal variation and the gender of the individual. This study showed that the PBMC isolation method by negative selection using the Robosep^TM^-S system is the best approach to measure CSA parameters. The problems with the OptiPrep PBMC isolation method might be due to the activation of Opti-isolated PBMCs, the presence of impurities and/or technical inconsistencies. The presence of platelets in Opti-isolated PBMCs but not in Robo-isolated PBMCs when using the Sysmex hemoanalyzer assay further confirms the problem of impurities in the case of Opti-isolated PBMCs. Furthermore, the normal RQ data distribution in Robo-isolated PBMCs according to the Shapiro–Wilk test (W ≈ 1) but not in the case of Opti-isolated PBMCs (Shapiro–Wilk 0.71 to 0.76) clearly demonstrates the technical inconsistencies in the case of the OptiPrep method of PBMC isolation. In addition to the PBMC isolation method, age, seasonal variation and the gender of the individual influenced the measurement of CSA parameters in PBMCs.

The most important CSA parameters influenced by these factors were maximal respiration (including spare capacity) and extracellular acidification. Parameters such as proton leak, non-mitochondrial respiration and coupling efficiency showed no or only weak differences. Nevertheless, it must be emphasized that proton leak is an especially useful parameter, as some patients showed abnormally high or very low proton leaks. Proton leak is also an indicator of metabolic fitness, as confirmed by our observation that the proton leak was much higher in warm seasons compared to cold seasons. This makes proton leak a very volatile parameter. The reason for this phenomenon might be the nature of the proton leak. In addition to the fact that proton leak is the consequence of damage to the mitochondrial membrane or proteins of the respiratory chain, proton leak is also caused by passive proton influx via adenine nucleotide translocase (ANT) or uncoupling proteins [38]. The same holds true for non-mitochondrial respiration. In PBMCs, non-mitochondrial respiration is the result of cell surface respiration, which is related to normal PBMC function [39,40]. Higher non-mitochondrial respiration might be an indicator of the activation of immune cells and, therefore, in turn, an indicator of inflammation or cellular stress.

Our study also showed that some of the main CSA parameters, such as spare capacity, extracellular acidification and mitochondrial respiration, increased with aging. This phenomenon can be explained in the following way: With aging, metabolism becomes slower, and thus, less ATP is consumed compared to younger individuals [31,32]. Nevertheless, more ATP is used for mechanisms that counteract age-related transformations within the cell, such as increased ROS formation, and the quality of mitochondria decreases [31]. As a result, ATP is generated more via non-mitochondrial mechanisms, such as anaerobic glycolysis, resulting in increased lactate formation. This further leads to an oversupply of reducing equivalents. By applying FCCP to the cells, enough reducing equivalents are available, and thus, mitochondrial respiration in the form of maximal respiration and spare capacity is higher.

In this study, mitochondrial respiration increased from colder months to warmer months, but extracellular acidification decreased. The opposite results were observed when warmer periods shifted to colder months. The tendency to increase in maximal respiration and spare capacity during summer indicates that both parameters are related to human well-being. The best physical performance according to Dhahbi et al.’s report was found in autumn [34]. Nevertheless, the study by Dhahbi et al. was conducted in Tunisia, where the weather is often warmer compared to Germany. The spare capacity in Opti-isolated PBMCs was almost constant from July to October and then dropped from November until March. This is in line with the observation made earlier concerning seasonal variations in vitamin D levels [41]. Other authors have argued that there might be a metabolic clock throughout the year (circannual rhythms), especially in colder and darker countries [42]. Moreover, physical activity is reduced in colder periods of the year [43], which is considered to lower mitochondrial function [31].

Moreover, in colder periods of the year, PBMCs are often challenged with infections and other metabolic transformations [33,41]. This leads to higher ATP consumption, and the ATP level declines. Because of the lower amount of ATP, more reduction equivalents are directly used for ATP generation. This leads to a lower supply of reducing equivalents, which are compensated by additional mechanisms, such as anaerobic glycolysis, and acidification increases. In warmer periods, however, PBMCs do not face problems as in colder months. Therefore, the pool of reducing equivalents is sufficient and available for mitochondrial respiration, especially maximal respiration. These findings are further supported by the fact that basal respiration was higher in Opti- and Robo-isolated PBMCs in colder months, indicating the permanent use of reducing equivalents. In contrast, all other respiratory parameters, including maximal respiration and spare capacity, were lower in colder months. This indicates that constant high oxygen consumption exists in winter and while aging.

In this study, we also observed that females tended to have lower extracellular acidification than males, with the largest difference in late adulthood (60 to 70 years). Moreover, old females showed a higher relative spare capacity than old males. These observations imply that the lower extracellular acidification and higher spare capacity in females might be the reason why females have a higher life expectancy than males [36], and these parameters could be considered as potential indicators of longevity. A previous report showed that females who were premenopausal appeared to have lower levels of oxidative stress compared to men. One reason for this apparent gender difference could be the antioxidant properties of estrogen. Moreover, antioxidant enzyme activity levels, NADPH-oxidase levels (especially p47 and Nox levels) and angiotensin II may also play important roles [35].

The limitation of this study is the lack of an analysis of the molecular mechanisms of cellular stress. However, the findings of this descriptive study are strengthened by assessing a wide range of cellular stress parameters related to both mitochondrial respiration and extracellular acidification.

## 4. Materials and Methods

### 4.1. Study Design, Period and Settings

A cross-sectional comparative study was conducted among patients and healthy individuals who visited hospitals in Germany, and blood samples were sent to the Magdeburg Molecular Diagnostics (MMD) laboratory for cellular stress assays from October 2017 to February 2020.

### 4.2. Study Participants

A total of 518 study participants (493 patients and 25 apparently healthy individuals) were enrolled in this study. For this comparative study, specific disease conditions were not targeted, and accurate pre-diagnosis was not required for the patient participants. For age-related comparisons, we defined five groups: young (12 to 35 years old), midlife (36 to 50 years old), mature adulthood (51 to 60 years old), late adulthood (61 to 70 years old) and old (71 to 90 years old). Similarly, both male and female participants were recruited during the four seasons of the year, namely, spring, summer, autumn and winter, for comparison purposes.

### 4.3. Blood Sample Collection

Following an aseptic technique, up to 16 mL of venous blood was collected from each participant in a vacutainer tube with Citrate-phosphate-dextrose solution with adenine (CPDA) anticoagulant. The blood samples were transported to the MMD laboratory immediately, and PBMCs were isolated for CSA within 24 h of blood collection. PBMCs were isolated by density centrifugation and negative selection methods. To exclude the potential influence of changes in the anticoagulant used and the proton concentration in the medium, we used the same anticoagulant and medium throughout the study. Prior to the study, we evaluated the influence of different anticoagulants, such as heparin, EDTA, ACD and CPDA, on CSA parameter results. PBMCs isolated immediately after blood collection showed similar CSA results regardless of the anticoagulant used. However, PBMCs isolated 24 h and 48 h after blood collection showed reliable results only in blood samples collected with the CPDA anticoagulant. The effect of temperature changes during the cell isolation process was minimized by following standardized isolation procedures.

### 4.4. PBMC Isolation by OptiPrep^®^ Method

PBMCs were isolated by density centrifugation using OptiPrep^®^ (Stemcell^TM^ technologies, Vancouver, Canada) (Opti-isolated). About 16 mL of whole blood (CPDA) was transferred to a 50 mL Falcon tube and filled up with 16 mL of RPMI-1640 (Sigma Aldrich, St. Louis, Missouri, USA) supplemented with 10 mM glucose and 2 mM pyruvate, both purchased from CarlRoth^®^, Karlsruhe, Germany. After careful mixing by inverting the sample, 10 mL of OptiPrep^®^ (Axis-Shield, Dundee, United Kingdom) (1077 g/cm^3^) diluted with PBS (according to Dulbecco’s protocol) was used to underlay the solution. Subsequently, the underlaid blood sample was centrifuged for 20 min at 700 xrpm in a Sorvall™ LYNX™ centrifuge (ThermoFisher^TM^ Scientific, Waltham, MA, USA) without brake. After obtaining the gradient, PBMCs were carefully removed with a 1 mL pipette, and RPMI-1640 was added to a final volume of 10 mL; the suspension was carefully mixed and transferred to a fresh 15 mL Falcon. The obtained 10 mL PBMC solution was then overlaid on 5 mL of OptiPrep^®^ (1063 g/cm^3^) with a peqMATE Electronic Pipette Controller (peqlab, Erlangen, Germany) low gravity.

Subsequently, another centrifugation step (350× *g*, 15 min, without brake) was carried out for platelet removal. The whole soluble fraction was removed using a Pasteur pipette, and the cell pellet was diluted using 1 mL of RPMI-1640; the volume was brought to 5 mL with RPMI medium. After additional centrifugation (250× *g*, 10 min, full brake; washing step 1), the liquid was discarded, and the cell pellet was diluted and brought to a volume of 5 mL as described before in washing step 1. Eventually, 20 µL of the washed cells was mixed with 5 µL of Acridine orange (AO)/propidium iodide (PI) AO/PI (Nexcelom Bioscience) cell viability dye and counted using a Cellometer^®^ Vision (Nexcelom Bioscience, Lawrence, KS, USA). The cells were then centrifuged again (250× *g*, 10 min, full brake; washing step 2), the liquid was discarded, and the cell number was adjusted to a concentration of 2.5 × 10^6^ cells/mL. All isolation steps were carried out using a Herasafe^TM^ sterile bench from ThermoFisher^TM^ Scientific at room temperature.

### 4.5. PBMC Isolation by Robosep^TM^-S Method

PBMC isolation by negative selection using magnet beads was carried out with the Robosep^TM^-S system and the EasySep^TM^ direct Human PBMC Isolation Kit (StemCell^TM^, Vancouver, Canada) according to the manufacturer’s protocol. In most cases, 2 mL of whole blood was sufficient to achieve the necessary number of cells. The final PBMC solution obtained in RoboTM-S buffer was centrifuged (250× *g*, 10 min, full brake), and PBMCs were counted as described in the case of cells isolated via density centrifugation. All isolation steps, except for the Robo^TM^-S selection step, were carried out using a Herasafe^TM^ sterile bench from ThermoFisher^TM^ Scientific at room temperature.

### 4.6. Cellular Stress Assay

The cellular stress assay (CSA) was carried out with 250,000 PBMCs (100 µL) per well in triplicates using the Seahorse XFp Analyzer (Agilent^TM^, Santa Clara, CA, USA) or the Seahorse XF96 Analyzer (Agilent^TM^, Santa Clara, CA, USA) according to the manufacturer’s protocol. In brief: the CSA is a Seahorse-based assay with the sequential stepwise injection of various mitochondrial inhibitors. First, basal respiration is measured, and then oligomycin is added to inhibit mitochondrial ATP synthase. This leads to the interruption of electron transport and thus lower oxygen consumption. This step is followed by the injection of carbonyl cyanide 4-(trifluoromethoxy) phenylhydrazone (FCCP), which uncouples the mitochondrial proton gradient, enabling electrons to reduce oxygen again. After adding FCCP, maximal respiration can be measured. Finally, the co-administration of rotenone and antimycin A (RotAA) stops the mitochondrial respiratory chain.

Based on this procedure, the following CSA parameters were measured: basal respiration, non-mitochondrial respiration, proton leak, spare capacity, maximal respiration and the extracellular acidification rate (ECAR). The entire procedure for CSA is described in detail elsewhere [4]. In this study, we made the following modifications to the protocol: The medium of choice was RPMI-1640. The final concentrations of mitochondrial inhibitors within the wells were 3 µM oligomycin (purchased from Agilent and Sigma, St. Louis, MO, USA), 3 µM FCCP (purchased from Agilent and Sigma) and 5 µM rotenone/antimycin A (both purchased from Sigma). In addition to ECAR, the proton production rate (PPR) was calculated using the buffer capacity according to Mookerjee and Brand 2015 [21]. The respiratory quotient (RQ; CO_2_ produced/O_2_ consumed) was calculated according to Mookerjee and Brand 2015 [21] using the equation PPR/rate of mitochondrial oxygen consumption (total cellular oxygen consumption rate minus any oxygen consumption that is insensitive to specific inhibitors of mitochondrial electron transport).

### 4.7. Statistical Analysis

The CSA parameter data were analyzed with the software Wave 2.6.0 (Agilent^TM^) and further processed with Microsoft Excel. Then, the data were transferred to the R program, and statistical analyses were performed with the R (version 3.6.2) program and RStudio (version 1.2.5033). The following packages were used for analysis: tidyverse, ggpubr, Table 1, formattable, summarytools, psych, MASS, gmodels, lsr, broom and caret. To prevent the influence of large sample sizes on *p*-values, we used Cohen’s d test to determine the effect size of our observations [44]. The data distribution normality was tested using the Shapiro–Wilk test.

## 5. Conclusions

This study shows that PBMCs isolated with OptiPrep showed much higher extracellular acidification and higher mitochondrial respiration compared to Robo-isolated cells. The findings of this study confirmed the problem of impurities and technical inconsistencies in the case of PBMCs isolated by the OptiPrep method compared to the Robosep^TM^-S method. Higher maximal respiration and spare capacity, as well as higher extracellular acidification, were observed in older study participants than in younger participants. In winter, maximal respiration and spare capacity decreased. From spring, however, until the end of summer/early autumn, spare capacity and maximal respiration continuously increased. Elderly men had higher basal respiration, spare capacity and extracellular acidification than females in both Opti- and Robo-isolated PBMCs. In general, this study clearly demonstrates that the results of CSA parameters are seriously influenced by the PBMC isolation method, age, seasonal variation and gender. Therefore, we recommend that researchers and physicians properly interpret the results of CSA parameters in PBMCs by considering these potential factors. It is important for doctors evaluating CSA results to use separate CSA evaluation standards based on the isolation method, age, gender and season-dependent factors, which can be used for the diagnosis and management of health problems. Therefore, further studies to establish standards of CSA parameter results considering these factors are needed. Further studies considering additional factors, such as mitochondrial mass, should also be conducted to improve the measurement of CSA parameters for the assessment of the real mitochondrial fitness. To assess the cellular stress situation in PBMCs, both extracellular acidification and mitochondrial respiration should be taken into account.

## Figures and Tables

**Table 1:** Variations in the results of cellular stress assay (CSA) parameters in Opti-isolated PBMCs (*n* = 302) and Robo-isolated PBMCs (*n* = 216).

CSA Parameters	Opti-Isolated PBMCs			Robo-Isolated PBMCs			Cohen’s d *
	Mean (SD)	95% CI	Shapiro–Wilk Test	Mean (SD)	95% CI	Shapiro–Wilk Test	
Basal respiration (pmol/min)	39.84 (13.69)	38.30–41.38	0.95	29.37 (6.76)	28.50–30.30	0.99	0.92
Basal OCR (pmol/min)	57.56 (17.92)	55.54–59.58	0.96	42.88 (8.54)	41.70–44.00	0.99	0.99
Non-mitochondrial respiration (pmol/min)	17.70 (6.10)	17.01–18.39	0.98	13.66 (4.33)	13.10–14.20	0.99	0.74
Non-mitochondrial respiration (%)	31.16 (7.53)	30.31–32.01	0.95	31.74 (8.56)	30.60–32.90	0.97	0.07
Coupling efficiency (%)	87.74 (9.00)	86.72–88.76	0.87	83.66 (10.18)	82.30–85.00	0.96	0.43
Proton leak (pmol/min)	5.11 (3.10)	4.76–5.46	0.92	5.14 (3.48)	4.68–5.60	0.89	0.01
Proton leak (%)	12.99 (7.21)	12.18–13.80	0.92	16.89 (9.47)	15.60–18.20	0.95	0.47
Maximal respiration (pmol/min)	141.83 (47.93)	136.42–147.24	0.98	120.75 (44.00)	115.00–127.00	0.96	0.46
Spare capacity (pmol/min)	102.00 (38.15)	97.70–106.30	0.98	81.22 (40.24)	85.90–96.60	0.97	0.28
Spare capacity (%)	263.45 (79.82)	254.45–272.45	0.98	312.60 (114.46)	257.00–328.00	0.98	0.51
Bioenergetic Health Index (BHI)	1.64 (0.31)	1.61–1.67	0.97	1.56 (0.36)	1.51–1.61	0.96	0.27
Basal ECAR (mpH/min)	15.76 (6.86)	14.99–16.53	0.89	7.01 (2.27)	6.71–7.31	0.94	1.61
ECAR oligomycin (mpH/min)	22.61 (9.52)	21.54–23.68	0.90	11.05 (3.31)	10.60–11.50	0.98	1.52
Maximal ECAR (FCCP) (mpH/min)	32.25 (10.74)	31.04–33.46	0.95	19.58 (4.85)	18.90–20.20	0.98	1.44

CSA = cellular stress assay; PBMCs = peripheral blood mononuclear cells; OCR = oxygen consumption rate; ECAR = extracellular acidification rate; FCCP = carbonyl cyanide 4-(trifluoromethoxy) phenylhydrazone; * = Cohen’s d result shows the effect size of the two PBMC isolation methods and is interpreted as d > 0.2 = small effect, d > 0.5 = medium effect, and d > 0.8 = strong effect; SD = standard division; CI = confidence interval.

**Table 2:** Cellular stress assay parameters related to proton production rate and respiratory quotient in Opti-isolated (*n* = 293) and Robo-isolated (*n* = 185) PBMCs.

CSA Parameter	Opti-Isolated PBMCs			Robo-Isolated PBMCs			Cohen’s d *
	Mean (SD)	95% CI	Shapiro–Wilk Test	Mean (SD)	95% CI	Shapiro–Wilk Test	
Basal PPR (pmol/min)	100.07 (41.98)	95.26–104.88	0.90	49.11 (1.155)	47.01–51.21	0.94	1.72
PPR Oligomycin (mpH/rnin)	140.29 (58.08)	131.64–146.94	0.91	72.21 (20.29)	69.29–75.13	0.98	1.56
Maximal PPR (FCCP) (mpH/min)	157.18 (66.41)	189.69–204.67	0.96	121.66 (29.57)	117.40–125.92	0.97	0.69
Basal RQ	1.77 (1.76)	1.68–1.86	0.71	1.14 (0.27)	1.10–1.18	0.92	0.50
Maximal RQ	1.27 (0.44)	1.22–1.32	0.76	0.92 (0.25)	0.88–0.96	0.82	0.98

CSA = cellular stress assay; PBMCs = peripheral blood mononuclear cells; PPR = proton production rate; RQ = respiratory quotient; FCCP = carbonyl cyanide 4-(trifluoromethoxy) phenylhydrazone; * = Cohen’s d result shows the effect size of the two PBMC isolation methods and is interpreted as d >0.2 = small effect, d > 0.5 = medium effect, and d > 0.8 = strong effect; SD = standard division; CI = confidence interval.

**Table 3:** Mean, standard deviation and median of spare capacity (pmol/min), spare capacity (%) and maximal respiration (pmol/min) by age group in Opti-isolated PBMCs (*n* = 302) and Robo-isolated PBMCs (*n* = 216).

PBMC Isolation Method	CSA Parameters	Age Group						
		Young (*n* = 40)	Midlife (*n* = 72)	Mature Adulthood (*n* = 77)	Late Adulthood (*n* = 64)	Old (*n* = 49)	Overall (*n* = 302)	Cohen’s d * (Young vs. Old)
Opti-isolated PBMCs	Spare capacity (pmol/min)							0.6
	Mean (SD)	86.7 (32.0)	96.0 (33.8)	105 (38.0)	111 (46.2)	106 (33.4)	102 (38.1)	
	Median (Min, Max)	78.1 (25.5, 160)	95.8 (4.73, 191)	104 (25.7, 2819)	111 (16.7, 218)	105 (36.2, 211)	100 (4.73, 281)	
	Spare capacity (%)							0.16
	Mean (SD)	251 (66.3)	255 (76.6)	265 (77.0)	280 (94.2)	263 (77.8)	263 (79.8)	
	Median (Min, Max)	248 (117, 390)	254 (17.8, 476)	254 (102, 449)	268 (47.5, 529)	260 (18.5, 411)	255 (17.8, 529)	
	Maximal respiration (pmol/min)							0.65
	Mean (SD)	122 (392)	134 (41.5)	147 (50.3)	152 (57.2)	148 (403)	142 (47.9)	
	Median (Min, Max)	116 (38.4, 207)	133 (29.9, 251)	141 (4.10, 352)	151 (35.4, 279)	146 (71.7, 278)	138 (29.9, 3529)	
Robo-isolated PBMCs	CSA Parameters	Young (*n* = 21)	Midlife (*n* = 56)	Mature adulthood (*n* = 66)	Late adulthood (*n* = 48)	Old (*n* = 25)	Overall (*n* = 216)	
	Spare capacity (pmol/min)							1.01
	Mean (SD)	70.8 (31.1)	95.3 (39.5)	87.0 (42.1)	90.6 (34.4)	111 (46.2)	91.2 (40.2)	
	Median (Min, Max)	59.2 (34.4, 160)	87.6 (12.7.211)	87.9 (5.66, 219)	86.1 (37.9, 198)	102 (27.3, 218)	87.7 (5.66, 219)	
	Spare capacity (%)							0.71
	Mean (SD)	271 (72.1)	313 (113)	300 (116)	327 (111)	352 (140)	313 (114)	
	Median (Min, Max)	278 (144, 397)	313 (37.0, 784)	315 (35.8, 500)	314 (141, 710)	347 (95.1, 653)	311 (35.8, 784)	
	Maximal respiration (pmol/min)							1.01
	Mean (SD)	97.0 (36.4)	126 (43.2)	117 (45.4)	119 (38.0)	143 (49.0)	121 (44.0)	
	Median (Min, Max)	87.5 (47.1, 210)	119 (471, 252)	114 (25.2, 263)	117 (55.4, 237)	142 (55.8, 266)	118 (25.2, 266)	

CSA = cellular stress assay; PBMCs = peripheral blood mononuclear cells; young = 12 to 35 years old; midlife = 35 to 50 years old; mature adulthood = 50 to 60 years old; late adulthood = 60 to 70 years old; old = 70 to 90 years old; SD = standard division; * = Cohen’s d results interpreted as d > 0.2 = small effect, d > 0.5 = medium effect, and d > 0.8 = strong effect.

**Table 4:** Mean, standard deviation and median of basal ECAR (mpH/min), ECAR oligomycin (mpH/min) and maximal ECAR (FCCP) [mpH/min] by life phase in Opti-isolated PBMCs (*n* = 302) and Robo-isolated PBMCs (*n* = 216) and the results of Cohen’s d analyses for the comparison of CSA parameters in young and old persons.

PBMC Isolation Method	CSA Parameters	Age Group						
		Young (*n* = 40)	Midlife (*n* = 72)	Mature Adulthood (*n* = 77)	Late Adulthood (*n* = 64)	Old (*n* = 49)	Overall (*n* = 302)	Cohen’s d * (Young vs. Old)
Opti-isolated PBMCs	Basal ECAR (mph/min)							0.15
	Mean (SD)	14.7 (8.69)	14.3 (4.94)	16.4 (7.29)	17.2 (6.99)	15.8 (6.42)	15.8 (6.86)	
	Median (Min, Max)	13.0 (3.97, 50.3)	13.8 (5.67, 27.6)	14.6 (3.98, 45.5)	15.9 (5.64, 43.21)	14.5 (5.16, 36.2)	14.3 (3.97, 50.3)	
	ECAR oligomycin (mph/min)							0.27
	Mean (SD)	20.2 (10.0)	20.6 (7.23)	24.5 (11.9)	24.0 (8.45)	22.7 (8.61)	22.6 (9.52)	
	Median (Min, Max)	16.8 (5.26, 63.2)	19.6 (0.29, 40.6)	22.3 (8.17, 75.1)	22.9 (7.00, 50.6)	20.2 (5.64, 47.2)	20.7 (5.26, 75.1)	
	Maximal ECAR (FCCP) (mph/min)							0.27
	Mean (SD)	29.4 (11.2)	29.7 (8.55)	34.5 (12.7)	34.3 (10.2)	32 1 (9.68)	32.3 (10.7)	
	Median (Min, Max)	25.7 (8.68, 588)	27.6 (11.3, 53.7)	30.8 (132, 80.8)	33.7 (10.5, 60.1)	30.8 (02.7, 56.1)	30.2 (8.68, 80.8)	
Robo-isolated PBMCs	Basal ECAR (mph/min)							0.95
	Mean (SD)	5.88 (1.52)	7.15 (2.27)	6.84 (2.27)	7.14 (82.29)	7.87 (2.48)	7.01 (2.27)	
	Median (Min, Max)	6.11 (3.19. 8.89)	6.66 (3.13,17.7)	6.50 (2.50, 13.1)	6.75 (3.19, 14.5)	7.24 (4.91, 14.8)	6.63 (2.50, 17.7)	
	ECAR oligomycin (mph/min)							1.24
	Mean (SD)	8.75 (2.36)	11.0 (3.38)	11.1 (3.21)	11.5 (3.44)	12.2 (3.17)	11.0 (3.31)	
	Median (Min, Max)	8.61 (4.90, 14.0)	10.2 (5.32. 21.9)	11.5 (3.42, 19.49)	11.4 (4.44, 20.7)	11.8 (6.88, 20.5)	10.8 (3.42, 21.9)	
	Maximal ECAR (FCCP) (mph/min)							1.13
	Mean (SD)	16.4 (3.65)	20.1 (4–98)	19.6 (4.85)	19.4 (4.68)	21.3 (4.82)	19.6 (4.85)	
	Median (Min, Max)	16.6 (8.90, 24.5)	19.5 (10.4, 31.81）	19.4 (5.91, 34.0)	18.4 (9.82. 31.719)	20.8 (11.8, 31.7)	19.2 (5.91, 34.0)	

CSA = cellular stress assay; PBMCs = peripheral blood mononuclear cells; ECAR = extracellular acidification rate; FCCP = carbonyl cyanide 4-(trifluoromethoxy) phenylhydrazone; young = 12 to 35 years; midlife = 35 to 50 years; mature adulthood = 50 to 60 years; late adulthood = 60 to 70 years; old = 70 to 90 years; d > 0.2 small effect; d > 0.5 medium effect; d > 0.8 strong effect; SD = standard division; CI = confidence interval; * = Cohen’s d results interpreted as d > 0.2 = small effect, d > 0.5 = medium effect, and d > 0.8 = strong effect.

**Table 5:** Mean, standard deviation and Cohen’s d of all relevant parameters after comparing warm and cold periods of the year in Opti- and Robo-isolated PBMCs.

CSA Parameters	Opti-Isolated PBMCs			Robo-Isolated PBMCs		
	Mean (SD)		Cohen’s d	Mean (SD)		Cohen’s d
	Warm (*n* = 55)	Cold (*n* = 138)		Warm (*n* = 78)	Cold (*n* = 44)	
Basal respiration (pmol/min)	33.64 (9.6)	40.99 (15.15)	0.53	30.72 (6.56)	26.55 (6.76)	0.63
Basal OCR (pmol/min)	48.43 (13.11)	59.44 (19.09)	0.63	43.92 (8.5)	40.64 (9.47)	0.37
Non-mitochondrial respiration (pmol/min)	14.79 (4.7)	18.5 (5.99)	0.66	13.35 (4.42)	14.09 (5.53)	0.15
Non-mitochondrial respiration (%)	30.71 (6.02)	31.83 (8.33)	0.14	30.12 (8.5)	34.31 (10.51)	0.45
Coupling efficiency (%)	85.95 (9.95)	88.54 (9.25)	0.27	80.58 (10.01)	87.51 (10.67)	0.68
Proton leak (pmol/min)	5.14 (2.68)	5.05 (3.52)	0.03	6.12 (3.78)	3.78 (2.79)	0.68
Proton leak (%)	15.06 (6.77)	12.26 (7.48)	0.38	19.56 (10.12)	13.81 (8.94)	0.59
Maximal respiration (pmol/min)	140.26 (44.14)	135.09 (44.34)	0.12	129.42 (40.88)	86.72 (29.56)	1.15
Spare capacity (pmol/min)	106.62 (36.81)	94.1 (32.82)	0.37	98.7 (36.96)	60.16 (29.23)	1.12
Spare capacity (%)	317.89 (71.56)	239.13 (68.34)	1.14	321.28 (102.81)	249.25 (135.07)	0.62
Bioenergetic Health Index (BHI)	1.65 (0.29)	1.62 (0.31)	0.10	1.52 (0.36)	1.49 (0.4)	0.08
Basal ECAR (mpH/min)	11.98 (4.22)	17.58 (8.13)	0.77	7.24 (2.67)	7.14 (2.05)	0.04
ECAR oligomycin (mpH/min)	16.18 (5.39)	25.11 (11.12)	0.91	10.82 (3.49)	11.57 (3.11)	0.22
Maximal ECAR (FCCP) (mpH/min)	25.77 (6.92)	34.25 (11.9)	0.79	19.58 (4.85)	20.02 (4.3)	0.20
Basal PPR (pmol/min)	76.27 (26.46)	111.39 (48.55)	0.81	49.81 (16.41)	50.17 (14.35)	0.08
PPR oligomycin (mpH/rnin)	100.58 (33.83)	155.48 (66.09)	0.93	70.78 (21.29)	76.75 (20.05)	0.28
Maximal PPR (FCCP) (mpH/min)	155.75 (43.77)	209.13 (70.87)	0.83	119.36 (29.97)	125.9 (26.83)	0.23
Basal RO	1.58 (0.37)	1.95 (0.94)	0.45	1.14 (0.28)	1.25 (0.26)	0.42
Maximal RQ	1.02 (0.2)	1.42 (0.52)	0.89	0.88 (0.22)	1.32 (0.35)	1.63

CSA = cellular stress assay; PBMCs = peripheral blood mononuclear cells; OCR = oxygen consumption rate; ECAR = extracellular acidification rate; PPR = proton production rate; RQ = respiratory quotient; FCCP = carbonyl cyanide 4-(trifluoromethoxy) phenylhydrazone; Cohen’s d >0.2 = small effect; d >0.5 = medium effect; d >0.8 = strong effect; SD = standard division; CI = confidence interval.

## Data Availability

The data presented in this study are available on request from the corresponding author. All relevant data are included in the paper.
